# Causal Inference in Clinical Research: A Primer

**DOI:** 10.1111/jre.70142

**Published:** 2026-07-17

**Authors:** Nasir Z. Bashir, Moritz Kebschull, Stephen Burgess, Gustavo G. Nascimento

**Affiliations:** ^1^ MRC Biostatistics Unit University of Cambridge Cambridge UK; ^2^ School of Dentistry University of Birmingham Birmingham UK; ^3^ Birmingham NIHR Biomedical Research Centre University of Birmingham Birmingham UK; ^4^ Division of Periodontics, Columbia College of Dental Medicine Columbia University New York New York USA; ^5^ Cardiovascular Epidemiology Unit University of Cambridge Cambridge UK; ^6^ School of Dentistry University of Utah Salt Lake City Utah USA

**Keywords:** causality, directed acyclic graph, epidemiology, periodontal diseases, randomized trial, study design

## Abstract

The study of causal relationships is central to scientific inquiry. Understanding what disease is, why it arises, and how it can be treated are a set of fundamentally causal questions. In this article, an overview of causal inference is provided for the applied researcher, with an emphasis placed on intuition and the use of examples from periodontology to support understanding. Causal reasoning goes beyond the description of associations, with a focus instead placed on questions of the form: what would happen to an outcome if a particular exposure were changed? Directed acyclic graphs are introduced as tools through which assumptions can be made explicit and structures that may threaten valid causal inference can be identified, including confounding, mediation, and collider pathways. Randomized controlled trials are highlighted as the gold standard of causal evidence, primarily because they ensure that, on average, comparisons are made between like‐with‐like groups. However, observational data must often be relied upon due to ethical, practical, and logistical constraints. A three‐step framework to answering causal questions is presented, based on (i) asking a causal question, (ii) identifying the causal effect, and (iii) carrying out the analysis. Within this context, two principal strategies for causal identification are outlined: design‐based identification and adjustment‐based identification. It is illustrated that each approach relies on different sets of assumptions to connect observed data to causal effects. The analysis stage carries out a pre‐defined associational comparison that is taken to have a causal interpretation under the proposed assumptions. Across all approaches, a central theme is that causal inference is inherently driven by assumptions. The credibility of a causal claim is determined not primarily by the statistical method, but by whether the underlying assumptions are considered plausible within the context of the scientific question.

## Graphical abstract

Causal inference helps researchers move beyond describing associations to answering the question: what would happen if an exposure were changed? This article provides an intuitive overview for applied researchers.

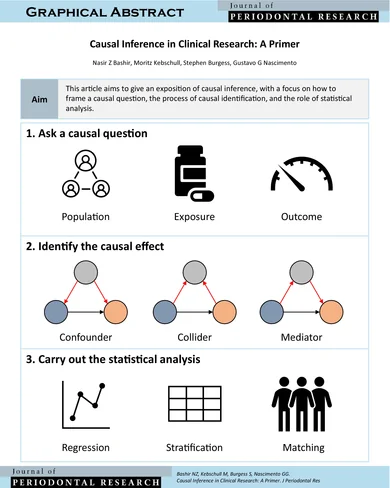

## 1 The Benefits of Bookshelves

A study is carried out, and it is found that individuals without periodontal disease own a greater number of bookshelves than those with disease. The study is repeated several times, and this association does not appear to be a chance artifact—it is observed repeatedly over the span of multiple years. The data used to inform these analyses are robust, involving large sample sizes that are representative of the entire population. The question must then be asked: do bookshelves protect individuals against periodontitis?

Of course, it is known that bookshelves do not provide protection against periodontitis. Despite the use of high‐quality data and carefully considered statistical methods, the conclusions drawn are somehow still inappropriate. Upon further consideration of this problem, it is revealed that those individuals who own bookshelves are likely to be of a higher socioeconomic position (SEP). That is, in this context, book ownership can be viewed as a proxy for SEP. For example, individuals who own more bookshelves are likely to have a higher income, which allows them to afford larger houses, more books, and also regular access to dental care. Or they may possess a higher level of educational attainment, which increases their propensity for reading and also increases their awareness of preventive oral hygiene habits. As a result, a reduction in the risk of having periodontitis is observed among those who own more bookshelves. It is said that the relationship between periodontitis and bookshelf ownership is *confounded* by SEP. In other words, if it were not for SEP, the association between periodontitis and ownership of bookshelves would not be observed.

The presence of confounding is a challenge that must be addressed in virtually all disciplines of science. Through the scientific method, investigators wish to answer questions such as “why” and “how” variables affect one another. These questions are fundamentally causal in nature and, as highlighted above, confounding obscures causality. Therefore, the ability to draw correct inferences hinges on whether causality can be distinguished from confounding.

Naturally, a seemingly simple question arises—what is a “cause”? The phrase “seemingly simple” is used because, in reality, the literature pertaining to this line of inquiry is vast. The Oxford English Dictionary defines a cause as “that which produces an effect”, a rather circular definition, as one is now left questioning what an “effect” is. The Cambridge Dictionary is (marginally) more informative, defining a cause as the reason why something happens. Note the philosophical, not mathematical, nature of these definitions. Much of the study of causality has been through a philosophical lens, and there are numerous schools of philosophy that present somewhat orthogonal definitions of causality [1].

Here, a pragmatic definition is adopted. A cause is defined as something that changes an outcome when it is perturbed [2, 3, 4]. To relate back to the initial example, changing an individual's SEP will alter their risk of periodontitis, but changing the number of bookshelves they own will not (assuming all else is held equal). Therefore, it is concluded that SEP has a causal effect on periodontitis, whilst bookshelves do not. This definition is useful because it allows for precise mathematical formulations of causal effects, which then facilitates their estimation. By no means is it being claimed that such a definition is perfect—for example, it is not specified *how* SEP is changed, nor is it considered whether it is even *possible* to change SEP. It is simply stated that, if it were changed, there would be a causal effect on the outcome. It should also be noted that temporality is inherent to this definition, i.e., a cause must precede its effect. This is accepted as a necessary, but not sufficient, condition for causality [5].

Despite being incredibly thought‐provoking, arguments pertaining to the definition of causality are not explored any further here; the line between epidemiology and philosophy becomes blurred [1].

## 2 Thinking Causally

### 2.1 Counterfactuals and Potential Outcomes

It might be assumed that causal reasoning is simple and intuitive, since questions around cause‐and‐effect are ubiquitously of interest to scientists [6]. However, causal assumptions are often kept implicit and even the use of the word “causal” is deemed to be unacceptable in much of the modern scientific literature, including apprehension towards the term by authors, reviewers, and editors [6, 7, 8].

At the heart of causal inference is the counterfactual, or “what if”, question [4, 9]. For example, if a patient with periodontitis were successfully treated, how would their quality of life be, compared to the state it would have been in had they not received treatment? Similarly, if a heavy smoker with periodontitis had never smoked, what would their periodontal status look like today? These unobservable scenarios form the basis of what is known as the potential outcomes framework, in which every individual has at least two potential outcomes: one under exposure (e.g., untreated disease, or smoking) and one under non‐exposure (e.g., successful treatment, or non‐smoking) [2]. It is only ever possible to observe one of these outcomes for each person, never both. The central challenge of causal inference is therefore to estimate these missing counterfactuals using comparisons across groups that differ in their distribution of the exposure, while accounting for confounding factors that might otherwise distort the contrast. Therefore, the study of causation can be seen as conceptually distinct to the study of prediction alone (Box 1). Whilst counterfactual reasoning is foundational to the contemporary formulation of causal inference, this conceptualization has not been without criticism, and interested readers may wish to explore these debates elsewhere [10].

BOX 1 **Prediction versus causation.**

**Prediction**

- Aims to estimate what is likely to happen.
- Focus is on forecasting outcomes in new individuals or populations.
- Associations may be very informative for prediction even if they are non‐causal.
- Model performance is typically evaluated using metrics such as discrimination or calibration.
- Example: Having yellow fingertips (“smoker's fingers”) may be predictive of periodontitis.

**Causation**

- Aims to estimate what would happen under a change or intervention.
- Focus is on understanding whether changing an exposure would change an outcome.
- Exposures can be strongly associated with the outcome without being causal.
- Requires assumptions that allow for causal interpretation, particularly like‐with‐like comparisons.
- Example: Smoking cessation may reduce the risk of developing periodontitis in the future.

Terminology has been introduced that is commonplace in the causal inference literature, such as *counterfactuals* and *potential outcomes*, and will be referenced throughout. These are summarized more comprehensively in Table 1.

**TABLE 1:** Summary of key causal inference terminology.

Term	Explanation	Example
Average treatment effect (ATE)	The average causal effect of an exposure in the entire population.	On average, quitting smoking reduces PPD by 0.5 mm across all patients (purely hypothetical).
Average treatment effect on the treated (ATT)	The average causal effect among those who actually received the exposure.	Among current smokers who quit, PPD decreases by 0.8 mm on average (purely hypothetical)
Backdoor path	A non‐causal path from the exposure to the outcome that can confound the effect.	Sex affects both smoking behavior and periodontal health, creating a backdoor path between the two.
Causal effect	The change in the outcome caused by changing the exposure, assuming everything else is constant.	The reduction in probing pocket depths if a patient quits smoking.
Causal identification	The set of assumptions under which a causal estimand can be uniquely expressed in terms of observed data.	The average treatment effect is identifiable under assumptions of exchangeability, positivity, and consistency.
Collider	A variable caused by two or more variables; conditioning on it induces spurious associations.	Tooth loss is caused by both dental caries and periodontitis.
Conditional average treatment effect (CATE)	The average causal effect within a subgroup defined by covariates.	The effect of quitting smoking is stronger among males (1.0 mm reduction in PPD) versus females (0.4 mm reduction) (purely hypothetical)
Conditioning	Adjusting for or restricting on a variable in the analysis.	Stratifying by age in a study of smoking and periodontal health, or controlling for it in a multivariable model.
Confounder^a^	A variable that causes both the exposure and outcome, potentially misleading the analysis.	Cigarette smoking causes both periodontal and cardiovascular disease.
Correlation/association	A statistical association between two variables, which may or may not reflect a causal relationship.	People who own more bookshelves may have a lower prevalence of periodontitis, showing correlation without necessarily implying causation.
Consistency	The assumption that the effect of the exposure is the same, no matter how it is assigned.	Interproximal cleaning has the same effect whether it is done by the patient themselves or instructed by a clinician.
Counterfactual/Potential outcome	The outcome that would have occurred under a different exposure scenario.	What a smoker's periodontal health would have been had they never smoked.
Directed acyclic graph (DAG)	A visual diagram of assumed causal relationships between variables.	See Figures.
Direct effect	The portion of the exposure's effect on the outcome that is not mediated by another variable.	Smoking's effect on periodontal tissue destruction that occurs independently of systemic inflammation (See indirect effect).
Effect heterogeneity	Variation in the causal effect of an exposure across individuals or subgroups defined by covariates.	The effect of smoking cessation on periodontal health may be stronger in older individuals than in younger individuals.
Effect modification/Interaction^b^	When the effect of the exposure varies depending on another variable.	Smoking may have a different effect on periodontal health in younger adults versus older adults.
Estimand	The specific quantity or causal contrast that a study aims to estimate, defined by the population, exposure, outcome, and summary measure.	In a study of smoking and periodontal health, the estimand might be the average reduction if all smokers quit (the ATE), or the effect among current smokers who quit (the ATT).
Exchangeability	The assumptions that groups are comparable except for the exposure, meaning confounders are balanced.	Individuals randomized to each group of a trial have similar/balanced sociodemographic characteristics.
Exposure/Treatment	The variable whose effect on an outcome is being studied.	Cigarette smoking in a study of periodontal health.
Generalizability	The extent to which an effect estimated in a study sample applies to a broader target population.	A trial conducted in a clinic population shows that quitting smoking reduces PPD by 0.5 mm; generalizability asks whether this effect applies to all patients with periodontal disease in routine care.
Indirect effect	The portion of the exposure's effect on the outcome that occurs through a mediator.	Smoking increases systemic inflammation, which in turn worsens periodontal tissue destruction. Here, the effect of smoking through inflammation is the indirect effect.
Instrumental variable	A variable that affects the outcome only through its effect on the exposure.	A genetic variant whose only role is to code for CRP can only affect the outcome by modulating CRP levels.
Interference/Spillover/Network effects	When the treatment of one individual affects the outcome of another.	Adoption of better oral hygiene practice by one person may influence other household members to adopt similar behaviors, improving their periodontal outcomes.
Measurement error/Misclassification	Errors in measuring the exposure, outcome, or confounder, which can lead to biased causal estimates.	Self‐reported brushing frequency may not accurately reflect actual brushing behavior.
Mediator	A variable that lies on the causal pathway between an exposure and outcome.	The effect of SEP on periodontitis is mediated by ability to afford dental care.
Outcome	The variable on which the effect of the exposure is being studied.	Periodontal probing depth in a study of smoking.
Positivity	The assumption that each individual has a non‐zero chance of receiving each level of the exposure.	Some patients have the possibility to quit smoking or not; there are no “impossible” exposure categories.
Randomization	Assignment of an exposure randomly (stochastically) to break confounding.	Randomly assigning a new oral hygiene program to study its effect on periodontitis.
Representativeness	The degree to which a study sample reflects a specified population with respect to relevant characteristics.	A sample of periodontal patients drawn from a specialist clinic is not representative of all adults in the population.
Selection bias	Distortion of causal estimates due to non‐random selection of participants.	Including only systematically healthy, young, non‐smokers, who regularly attend the dentist.
Source population	The population from which the study sample is drawn.	Patients attending dental clinics in a given healthcare system form the source population.
Stable unit‐treatment value assumption (SUTVA)	Assumes no interference between individuals and no “hidden” versions of treatment.	One patient's periodontal outcome does not depend on whether another patient quit smoking.
Target population	The population to which causal effects are intended to generalize.	All adults in the general population with or at risk of periodontal disease.
Temporality	The requirement that the exposure precedes the outcome in time; a necessary but not sufficient condition for causal inference.	Onset of periodontitis cannot be caused by smoking in individuals who only started smoking after disease onset. However, even when smoking clearly occurs before disease onset, this temporal ordering alone does not rule out confounding or other sources of bias.
Transportability	The extent to which a causal effect estimated in one population can be validly transferred to a different (generally non‐overlapping) target population.	A smoking–periodontitis effect estimated in a specialist dental clinic is transported to the general population, assuming differences in disease severity and health behavior are appropriately accounted for.

Abbreviations: CRP, C‐reactive protein; PPD, Probing pocket depth; SEP, Socioeconomic position.

^a^ Note that there is some simplification here. The most precise definition of a confounder is a variable that is contained in a minimal sufficient adjustment set; however, the definition in terms of a common cause of the exposure and outcome is more useful for exposition here.

^b^ Note that there is some simplification here. Effect modification is a causal concept describing heterogeneity of effects, whereas interaction is a statistical concept that depends on the chosen scale of measurement.

### 2.2 Visualizing Causal Relationships

Directed acyclic graphs (DAGs) are an intuitive way to communicate and present causal relationships between variables [11]. A DAG is a visual representation of assumptions about how different variables in a system are related. It consists of nodes, which represent variables, and directed edges (arrows), which represent causal effects. The fact that it is “directed” means that the arrows encode a specific direction, from cause to effect. The term “acyclic” refers to the absence of feedback loops; if the arrows are followed forward, it is not possible to return back to the starting point [12].

Returning to the bookshelves example, DAGs can be used to make explicit the assumptions underlying different causal interpretations. First, the naïve assumption is made that bookshelf ownership directly causes a decrease in the risk of periodontitis (Figure 1). In reality, SEP acts as a confounder of these two variables, influencing both bookshelf ownership and the risk of periodontitis (Figure 2). A further compatible representation is to posit that increased knowledge of oral hygiene habits acts as a *mediator* of the relationship between SEP and periodontitis (Figure 3). That is to say, higher SEP increases knowledge of oral hygiene habits, which in turn reduces the risk of periodontitis.

**FIGURE 1:**
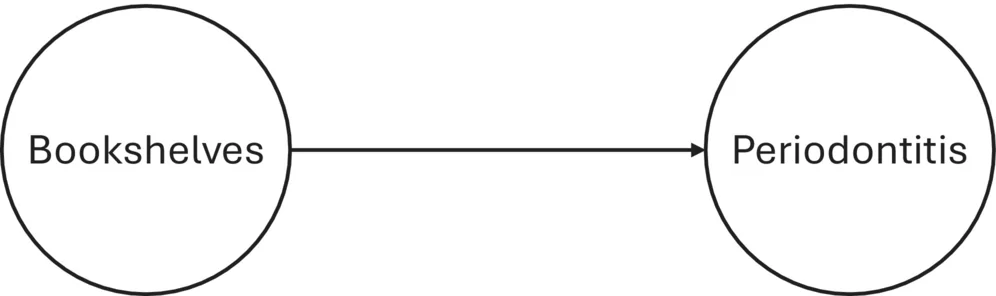
Directed acyclic graph showing the naïve assumption that bookshelf ownership is a causative agent for periodontitis.

**FIGURE 2:**
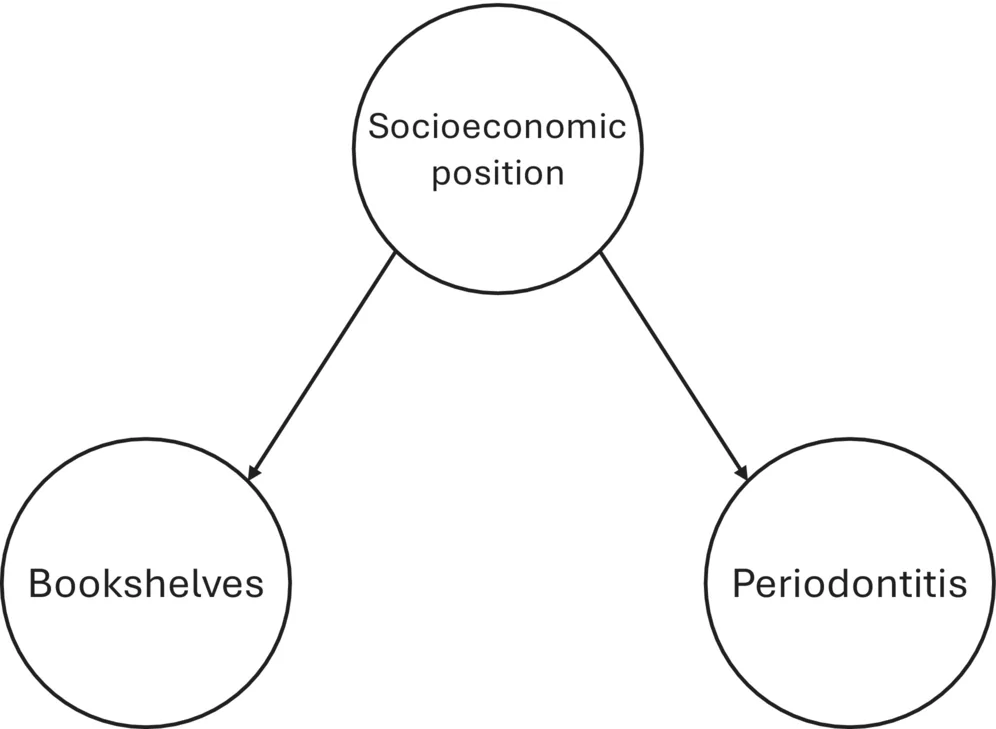
Directed acyclic graph showing socioeconomic status as the common cause (i.e., confounder) of bookshelf ownership and periodontitis.

**FIGURE 3:**
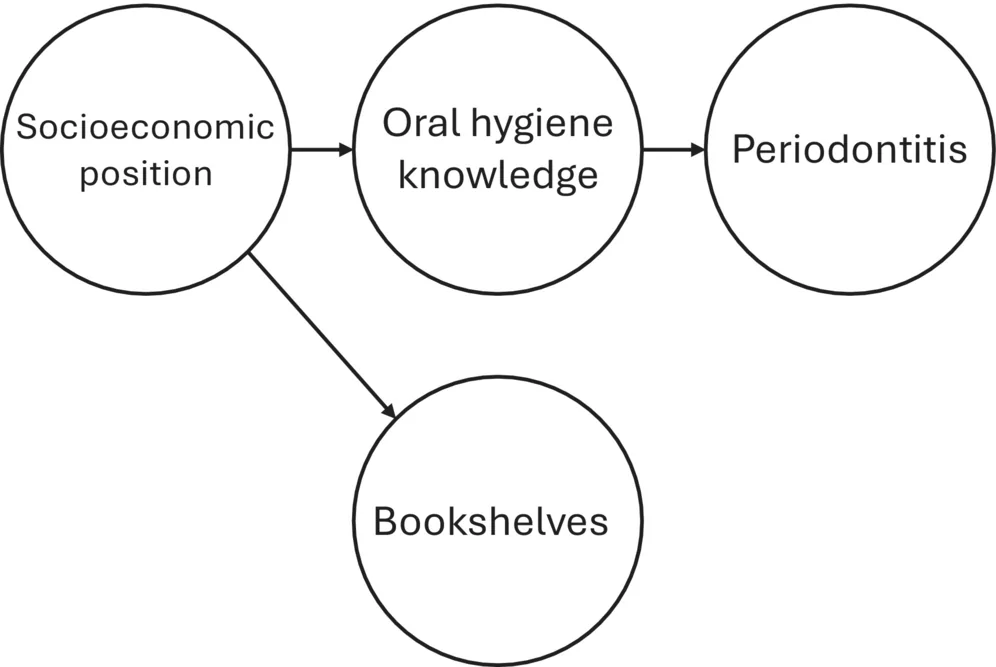
Directed acyclic graph showing knowledge of oral hygiene habits as a mediator between socioeconomic status and periodontitis.

Along with confounders and mediators, an important third type of variable is recognized, known as a *collider*. Consider the case where it is known that an individual has excellent plaque control with a relatively low bacterial load intraorally. It is then observed that this same individual has unstable periodontitis; upon learning this, it can be deduced that a very high probability of an aberrant immune response is present for this individual. The reason is that bacterial load and the host response are thought to be two of the major causes of periodontitis—if an individual with good plaque control experiences periodontitis, then it is highly likely that they must have a dysregulated immune response. In this situation, periodontitis is said to act as a collider i.e., it is caused by both plaque control and the host immune response (Figure 4).

**FIGURE 4:**
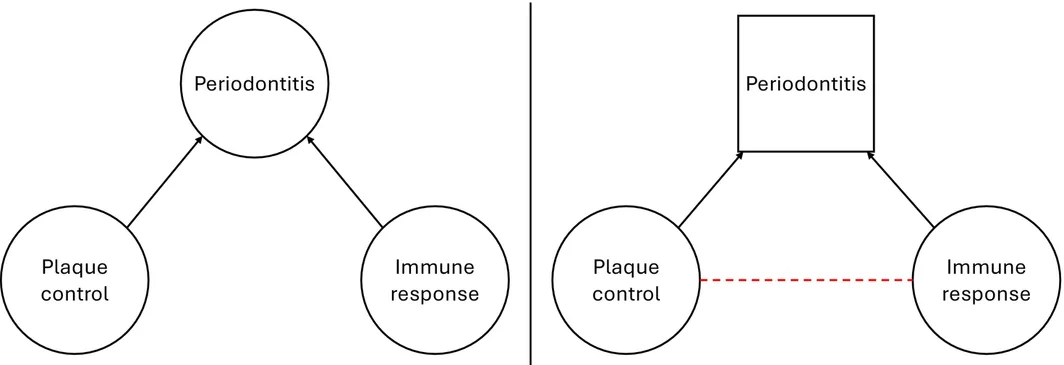
Directed acyclic graph showing periodontitis as a common effect (i.e., collider) of plaque control and dysregulated immune response. Square nodes denote variables that were conditioned on e.g., by restricting to individuals with periodontitis. Dashed red lines denote correlation between variables that are not necessarily causal. *Left*: Relationships between variables before conditioning on periodontal disease status. *Right*: Relationships between variables after conditioning on periodontal disease status. After conditioning on periodontal disease status, an association is induced between plaque control and dysregulated immune response.

Conditioning on a collider (e.g., by restricting the study sample to individuals with periodontitis, or by adjusting for it) can induce an association between its causes. This logic follows from the fact that, in a collider structure, the two causes become statistically dependent once the collider is conditioned on. Hence, knowledge of one cause (plaque control) provides information about the other cause (dysregulated immune response), even if the two share no direct causal connection. This phenomenon can lead to misleading inferences if it is not properly accounted for [13, 14].

Of course, there is room for disagreement in DAGs. It might be argued that periodontitis itself influences plaque control, for example, by inducing recession or increasing probing pocket depths, which make it more difficult to clean affected areas. Or there may be a feedback relationship, where periodontitis worsens the immune response, which in turn worsens periodontitis, and so on. Distinguishing between these possibilities requires careful attention to temporality (i.e., the order in which events occur), something that can be better established using longitudinal data [15]. Ultimately, however, the key issue is not disagreement over causal relationships, but rather the transparency of the assumptions underlying any proposed model. Clear communication of these assumptions allows researchers to appropriately interpret findings and avoid drawing misleading conclusions [16].

Therefore, the strength of DAGs lies in their ability to make assumptions transparent [17]. Investigators are forced to articulate which variables they believe cause others and, just as importantly, which variables are not believed to cause others. This is critical because causal inference depends on assumptions that cannot be proven from the data alone [4]. By committing these assumptions to a clear visual form, it becomes easier to spot potential sources of bias, and also to identify the set of variables that need to be controlled for in order to block all non‐causal pathways between an exposure and an outcome [18]. In other words, they help to decide what needs to be controlled for to obtain an unbiased estimate of the causal effect of interest, as well as what should not be controlled for in order to avoid introducing new biases.

While all DAGs are a simplification of reality, they nevertheless provide a shared language for reasoning about causality. They help ensure that scientific discussions, and eventual disagreements, about causality are grounded in explicit assumptions rather than vague descriptions.

### 2.3 A Framework for Causal Analysis

Now that the necessary background has been established, a framework for answering a causal question is outlined. Although the terminology and emphasis may vary across disciplines, most approaches can be understood in terms of three key elements (Figure 5):

1. Asking a causal question
2. Identifying the causal effect
3. Carrying out the statistical analysis

**FIGURE 5:**
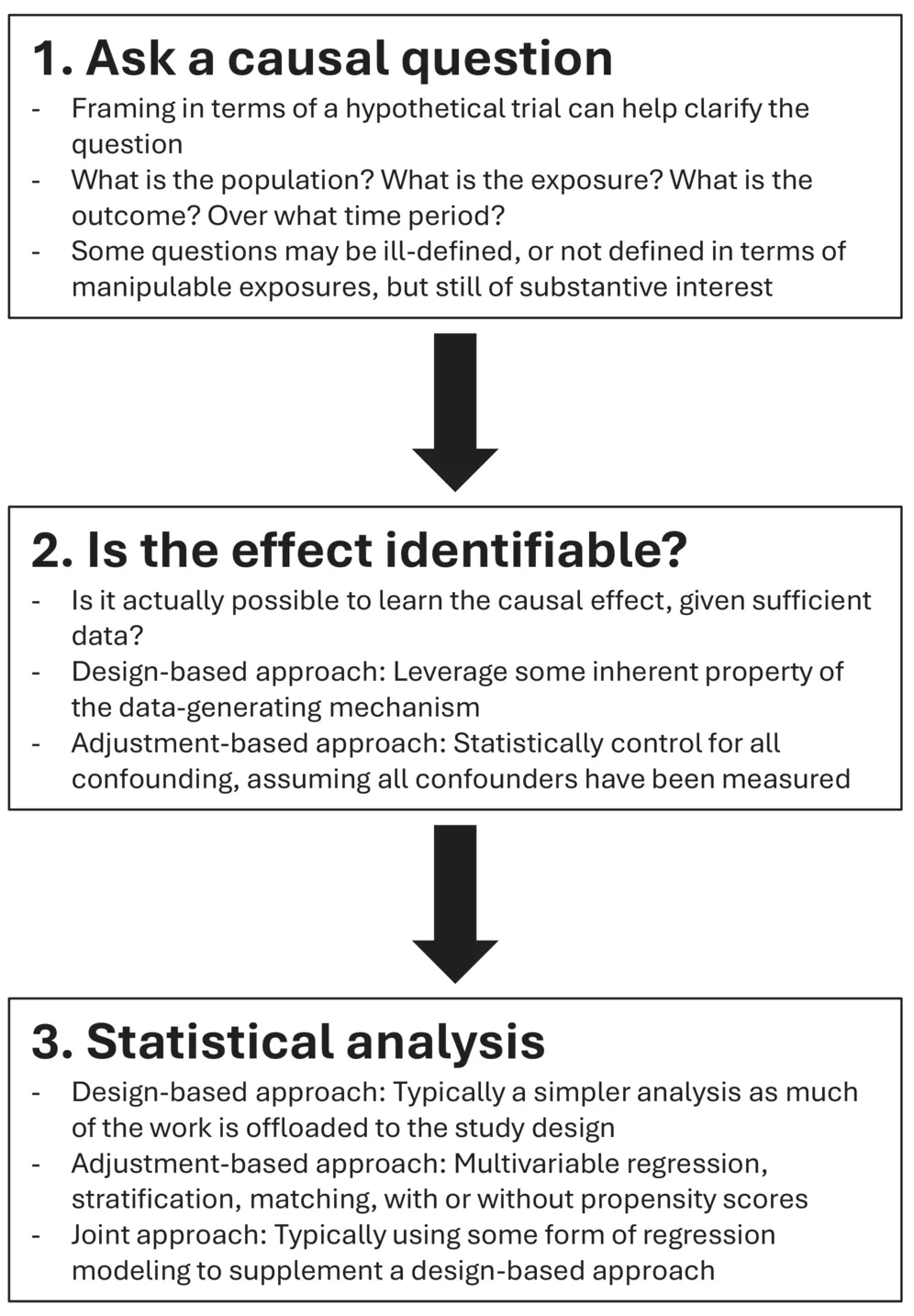
Schematic illustrating the key steps in answering a causal question.

These steps are presented sequentially, but in practice they are often iterative. For example, difficulties encountered during the analysis stage may reveal that the causal question was not sufficiently well‐defined, or that the assumptions required for identification are not plausible. In such cases, it is necessary to revisit earlier steps and refine the question or the underlying causal model.

## 3 Begging the Question

The first, and most central, step in causal reasoning is to ask a causal question [19]. Without a clearly specified question, it is not possible to determine what effect is being estimated, which assumptions are required, or whether the chosen analytical approach is appropriate. Despite this, the formulation of the causal question is often implicit, with greater emphasis placed on analytic methods than on the definition of the question itself.

A common misconception is that one must employ advanced statistical methods in order to frame and answer a causal question. This is not the case. Many studies in periodontology already use regression models that attempt to control for confounding by adjusting for factors such as age, sex, SEP, and smoking status [20, 21, 22, 23]. Nevertheless, authors frequently describe their study as being primarily interested in “associations”, for example, between smoking and periodontitis, or periodontitis and quality of life. If investigators were only interested in associations, then the task would be trivial: one could simply compute the correlation between periodontitis and the variable of interest, and the “association” would be established. The moment adjustment for confounders is introduced, it is implicitly acknowledged that mere association is unsatisfactory. Rather, the aim has shifted—perhaps implicitly—to uncover the effect that periodontitis has on downstream outcomes, or the effect of risk factors on periodontitis itself. In other words, the language of association often underplays the causal ambitions that researchers are already pursuing in practice.

Mislabeling questions of causation and association risks creating conceptual confusion. Readers may assume that results are purely descriptive when, in fact, the study design has made causal assumptions. Recognizing this makes clearer both the strength of the inferences that can be drawn and also the limitations when those assumptions may not hold. A concept which is central to the definition of causal questions, along with appropriate design and analysis of studies to answer said questions, is *randomization*.

### 3.1 What Is the Magic of Randomization?

The guiding principle of causal inference is to make *like‐with‐like* comparisons. That is, if two groups are being compared, the only thing that should differ between them is the exposure of interest, with all other factors held equal. When two (or more) groups can be compared in this way, they are said to be *exchangeable*. Exchangeability means that, aside from the exposure under study, the groups are comparable in all respects that could influence the outcome. Referring back to the bookshelf example, if individuals were divided into two groups according to whether they own bookshelves, any observed differences in periodontal status could not reasonably be attributed to the causal effect of bookshelf ownership itself. This is because the comparison is not like‐with‐like; the groups differ in more ways than just bookshelf ownership. For example, they may also differ in SEP. But, more broadly, groups may differ across many variables—potentially infinitely many—most of which the investigator may not be able to measure or even identify. This is precisely why confounding is such a central challenge in causal inference. Without exchangeability, observed associations cannot be straightforwardly interpreted as causal effects, because the groups being compared are not equivalent in all relevant ways, except for the exposure of interest.

Randomized controlled trials (RCTs) are widely regarded as the most reliable source of evidence [3, 24]. This privileged status arises from the fundamental property of randomization: by allocating individuals to treatment groups randomly, the distribution of measured and unmeasured confounders becomes balanced across the study groups, thus allowing the groups to be comparable [25]. In other words, randomization guarantees that groups are, on average, exchangeable. As a result, any difference in outcomes can be attributed to having been caused by the intervention. It is worth noting that randomization is so valuable because it ensures that groups do not systematically differ in their distribution of all confounders, both known and unknown. For most questions, researchers are unlikely to know all relevant confounders and, even if they do, it may be impossible to measure them all. Throughout, the term “unmeasured confounders” is used to include both unknown confounders and those that are known but not recorded.

Consider a hypothetical RCT where the aim is to understand the causal effect of cigarette smoking on periodontal health (recognizing from the outset that such a study would be ethically infeasible in practice). Typically, individuals who smoke differ from those who do not in a systematic manner; for instance, they may be older, more likely to be male, of lower SEP, and have different oral hygiene behaviors. These differences make direct comparisons between smokers and non‐smokers prone to extensive confounding. However, by randomly allocating individuals to smoke cigarettes, the association between smoking and these confounding factors is broken. Any differences in periodontal outcomes observed between the randomized groups could then be attributed to the effect of smoking itself, rather than to pre‐existing differences between participants. This illustrates why randomization is considered the gold standard for causal inference: it creates a situation where the counterfactual contrast (i.e., the hypothetical difference in outcomes if an individual did or did not smoke) can be approximated, provided the randomization is carried out adequately.

Note, however, that while randomization removes systematic differences between groups, some differences in measured or unmeasured characteristics may still arise by chance in any particular study. This is especially true when sample sizes are small, as randomization ensures that such chance differences become less likely and less consequential as sample size increases. This is why the guarantee of exchangeable groups is only true “on average”.

The DAG corresponding to an RCT is presented in Figure 6, where it can be seen that the exposure (intervention) is caused by randomized allocation. By definition, randomized allocation is independent of all confounders, which means that any confounding pathways between the exposure and outcome are blocked. In other words, the central identifying assumption of an RCT is that randomization balances confounders across treatment arms. This explains why the primary analysis of an RCT is often conducted as an intention‐to‐treat analysis, where participants are analyzed according to the group to which they were originally randomized, regardless of whether they adhered to the assigned intervention. This preserves the balance of confounders between the study groups—which maintains their exchangeability—ensuring that the analysis is unbiased [26]. For example, if non‐adherence to the intervention is determined by socioeconomic factors, then the group actually receiving the treatment may differ systematically from those who did not, thereby reintroducing confounding. The intention‐to‐treat principle addresses this problem by anchoring the analysis to the randomized allocation rather than actual treatment received. This preserves causal interpretability, but it estimates the effect of being offered treatment rather than the effect of actually receiving it. Whether that distinction is acceptable depends on the research question.

**FIGURE 6:**
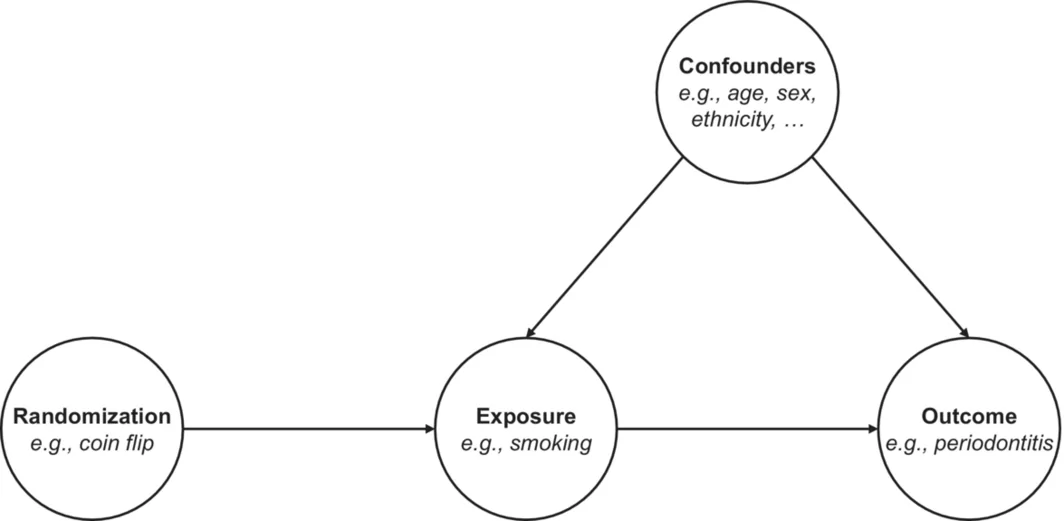
Directed acyclic graph representing the causal relationships in a hypothetical RCT investigating the effect of smoking on periodontitis. The node representing randomization corresponds to treatment allocation (e.g., a coin flip), whereas the smoking node represents actual exposure status. Confounders may influence smoking behavior but, by design, do not influence randomized allocation.

Despite their strengths, RCTs only go a small way in helping investigators to understand causal relationships [27]. First, it would be unethical and practically infeasible to conduct an RCT that randomly assigns individuals to smoke cigarettes. Second, even if such an RCT were permissible, interest may lie in the long‐term effects of smoking on periodontal health. In this case, it would be extremely challenging to ensure adherence to the assigned exposure over many years, and there may be substantial loss to follow‐up, meaning that periodontal outcomes cannot be observed. Third, even if it were permissible and adherence was guaranteed, the cost of carrying out such a study with large sample sizes may be prohibitively expensive. Finally, there are many exposures, such as ethnicity or childhood risk factors, which cannot be manipulated experimentally.

Because of these limitations, much of causal inference relies on observational data. The aim is to either emulate the comparison that would have been made in an ideal RCT, or to identify settings in which the exposure appears to be allocated “*as‐if*” at random [28, 29]. In observational data, where treatment is not randomly assigned, this generally requires identifying or approximating groups of individuals who are exchangeable, so that differences in outcomes can be interpreted causally.

It is worth noting that randomization is not a panacea for all aspects of trial conduct. Practical issues such as protocol deviations, informative drop‐out, lack of blinding, and missing data are important considerations in the design and analysis of randomized trials. However, these issues largely relate to the operational implementation of the trial rather than the core function of randomization, which is its ability to create exchangeable groups.

### 3.2 The Hypothetical Trial

To define a causal question precisely, it is helpful to imagine a hypothetical randomized trial that could answer it. This idea, sometimes referred to as the “target trial”, provides a concrete way of translating an abstract causal question into a well‐defined comparison [30]. Rather than beginning with the data that are available, the question is instead asked: *what experiment would ideally be run?* The purpose here is not to propose that such a trial is feasible, ethical, or practical. Instead, the hypothetical trial serves as a conceptual device. It forces the investigator to be explicit about the desired comparison they wish to estimate, for whom, and over what time period. In doing so, it clarifies the causal question independently of the data at hand.

To construct such a question, several components must be specified. First, the *population* of interest is defined. For example, investigators may be interested in adults with no history of periodontal disease at baseline. Second, the *intervention*, or *exposure*, is specified. In the context of smoking, this might correspond to assigning individuals to initiate cigarette smoking. Third, the *comparison* condition is specified, which is the alternative exposure state against which the intervention is evaluated. For example, abstinence from cigarette smoking. Fourth, the *outcome* is specified, such as the incidence of periodontitis over a given follow‐up period. Note that a definition of the time horizon over which outcomes are measured is usually required. Taken together, these elements define a precise causal contrast: what would be the difference in the risk of periodontitis if all individuals in the population were assigned to smoke compared to if all were assigned not to smoke over a specified period of time?

Importantly, different ways of specifying the hypothetical trial correspond to different causal questions. For instance, assigning individuals to “not smoke” is not the same as assigning them to “reduce smoking by half”, nor is it equivalent to “cessation after ten years of smoking”. Each of these interventions defines a distinct counterfactual scenario and, therefore, a different causal effect. Failure to clearly define the intervention can lead to ambiguity in interpretation. Within this hypothetical trial framework, causal questions are tied to interventions, whether real or imagined. This is consistent with the definition of a cause introduced earlier: a cause is something that changes an outcome when it is perturbed. In practice, the hypothetical trial is often not feasible. Nevertheless, observational studies can be designed and analyzed with the aim of estimating this, now well‐defined, causal effect as accurately and precisely as possible.

The hypothetical trial has been used here primarily as a conceptual framework for formulating a precise causal question. However, this idea can be extended beyond question formulation into a full analytic strategy known as *target trial emulation*. In target trial emulation, investigators do not simply imagine the ideal randomized trial—they explicitly attempt to reproduce its protocol using observational data. Thus, the hypothetical trial can be viewed at two levels. At its most fundamental level, it is a framework for sharpening the causal question. At another, it provides a foundation of target trial emulation, where the entire design and analysis of an observational study are organized around the goal of approximating that hypothetical experiment. Further details of methodological considerations in target trial emulation are available elsewhere in this journal [31].

### 3.3 The Ill‐Defined Question

The formulation of causal inference outlined above—a well‐defined contrast corresponding to a hypothetical trial—is one of the most widely used approaches in modern epidemiological research. In this framework, a causal question is only meaningful once the intervention, population, comparison, and outcome have been explicitly specified. Broadly speaking, this is the framework adopted throughout the present work.

The aim, however, is not to equate causal inference exclusively with this idealized formulation. In many areas of research, investigators begin with questions that are not immediately expressible as well‐defined interventions. For example, consider questions such as: “Does socioeconomic position affect periodontal health?”, “Is inflammation a cause of disease progression?”, or “What is the effect of genetic liability on periodontitis?” These questions are intuitively causal, yet they do not correspond directly to a precisely defined intervention. It is not obvious how one would randomize individuals to different levels of SEP, different levels of inflammation, nor how one would assign “genetic liability” in a conventional experimental sense. As such, the corresponding hypothetical trials are difficult to conceptualize. This highlights an important distinction. A question may be causal in intent without being causally well‐defined. Just because the intervention is not precisely defined, it does not mean SEP, inflammation, or genetic liability are immediately precluded as potential causative agents.

In settings such as these, investigators sometimes choose to work with more loosely defined contrasts. Mendelian randomization provides an instructive example [32]. In Mendelian randomization studies, genetic variants are used as proxies for modifiable exposures, under the assumption that these variants are randomly allocated at conception. Investigators often interpret Mendelian randomization estimates as reflecting the causal effect of an exposure (e.g., smoking or body mass index) on an outcome. However, the intervention corresponding to these genetic proxies is rarely made explicit. For instance, does a Mendelian randomization estimate for body mass index correspond to the effect of sustained weight loss, short‐term dietary change, or lifelong differences in adiposity? Each of these interpretations implies a different hypothetical intervention. Despite this, such analyses may still be informative for testing aspects of the underlying causal hypothesis. In particular, Mendelian randomization can be viewed as providing evidence on whether perturbations in a biological pathway, proxied by genetic variation, are capable of influencing the outcome. From this perspective, the goal is not necessarily to estimate the effect of a well‐defined intervention, but to assess whether a causal relationship is likely to exist at all [32].

Furthermore, some frameworks for causality do not rely on invoking a notion of intervention at all. A prominent example is Rothman's sufficient‐component cause model [5, 33]. In this approach, a disease outcome is understood as arising when a complete “causal mechanism”, or sufficient cause, is assembled. Each sufficient cause consists of a set of component causes—factors which, together, are enough to produce the outcome. No single component cause is necessarily sufficient on its own, but each may contribute as part of one or more sufficient causes. For example, the development of periodontitis might require some combination of microbial exposure, host inflammatory response, smoking, genetic susceptibility, and environmental or social conditions. Different individuals may reach the same outcome through different sufficient causes, meaning that multiple distinct causal pathways can lead to the same disease (Figure 7). Under this framework, asking whether SEP or inflammation is “a cause” does not require imagining an intervention that assigns individuals to different levels of these factors. Instead, the question is whether that factor functions as a component within one or more sufficient causes of the outcome. This perspective shifts attention away from estimating the effect of a specific intervention and toward understanding whether a factor plays a genuine etiologic role.

**FIGURE 7:**
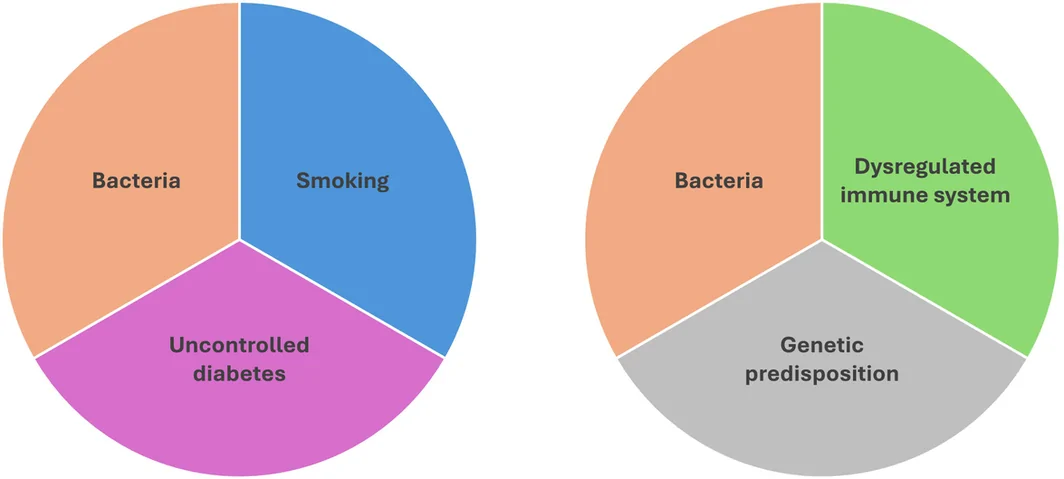
Rothman's sufficient‐component cause model illustrated using “causal pies”. Each pie represents a distinct sufficient cause for periodontitis in two different individuals. Each sufficient cause is composed of multiple component causes that together are sufficient to produce disease. In both pies, bacterial exposure is a necessary component cause, in that periodontitis does not occur in its absence. The remaining components differ across pies, illustrating that periodontitis can arise through multiple causal pathways, including combinations involving smoking, uncontrolled diabetes, dysregulated immune response, and genetic susceptibility.

For some exposures, particularly social variables such as race or SEP, a hypothetical intervention may be ill‐defined or inappropriate. Alternative frameworks, such as disparity decomposition methods, instead focus on explaining observed disparities, rather than defining a literal intervention on the exposure itself, illustrating that meaningful causal questions need not always be framed through a hypothetical trial [34, 35].

To summarize, the hypothetical trial framework provides a principled way to refine a causal question. This often involves iterating between substantive knowledge and formal reasoning. An initially broad question, such as the effect of smoking on periodontal disease, may be progressively refined into more specific contrasts, such as the effect of smoking initiation versus abstention, or the effect of cessation at a given age. Each refinement corresponds to a different causal estimand—that is, the exact causal effect one is trying to quantify from the data—and may require different assumptions and methods for identification. However, it is not argued that such a framework is the *only* valid way in which one may conceive of cause‐and‐effect relationships.

## 4 Identification

### 4.1 What Is Identification?

Once the causal question has been clearly defined, investigators will typically have a sense of the variables involved in the underlying causal hypothesis. The central challenge at this stage is to determine whether the causal effect of interest can be identified from the available data. Identification refers to whether it is possible to learn the causal effect given sufficient data under a proposed set of assumptions. Importantly, this is a conceptual step that precedes statistical analysis. It asks not *how* to estimate an effect, but whether it is *possible* to do so at all.

To address this, investigators must make their assumptions about the data‐generating process explicit, and DAGs are one useful framework for this purpose. Within this framework, identification typically involves determining whether all non‐causal paths between the exposure and outcome can be blocked by controlling for an appropriate set of variables. If such a set exists (and is measured without error), then the causal effect can, in principle, be identified from the data. Conversely, if important confounders are unmeasured, or if inappropriate variables (e.g., colliders) are controlled for, the resulting estimate may be biased, regardless of the statistical method employed.

Randomized controlled trials represent a special case in which identification is achieved by design. As discussed previously, randomization ensures that the exposure is independent of both measured and unmeasured confounders, thereby eliminating non‐causal pathways between the exposure and outcome. In observational studies, however, identification relies entirely on the plausibility of the assumptions encoded in the causal model. It is important to emphasize that identification does not arise from analyzing data; rather, it depends on a well‐specified causal structure. Statistical analysis becomes relevant only after it has been established that the causal effect is identifiable under the assumed model. This distinction highlights why careful causal reasoning, supported by transparent assumptions, is essential prior to any statistical analysis. In practice, identification is rarely guaranteed. Investigators must therefore consider whether their assumptions are credible in the context of the research question, and whether alternative strategies may be required to support causal interpretation.

The key message is that all methods for making causal claims rely on assumptions that connect the observed data to the outcomes that would have been seen under different exposure scenarios. Some assumptions can be assessed empirically, while others are fundamentally untestable and must be justified on substantive grounds. Understanding these assumptions is more important than memorizing the technical details of any one method. When attempting to infer causality, the central question is not “Which method is best?” but rather “Given the question and data at hand, are the assumptions of this method plausible?” An approach may yield credible results if its assumptions hold, but highly misleading results if they are violated. Thus, causal inference is less about identifying a universally superior method and more about developing a clear understanding of the conditions under which a method delivers valid causal conclusions.

To make these ideas more concrete, it is useful to consider two broad approaches to identification. These will be referred to as design‐based and adjustment‐based approaches. This is a useful dichotomization for exposition, but it is not definitive, as applied methods may combine elements of both.

### 4.2 Design‐Based Identification

Design‐based approaches obtain identification by exploiting features of the data‐generating process that induce variation in the exposure that is plausibly independent of confounders. In doing so, they aim to create a situation in which groups of individuals can be regarded as exchangeable. As previously established, exposure is rarely randomly assigned in observational data. However, design‐based approaches often make use of so‐called *exogenous* variation in exposure. Exogenous variation refers to changes in the exposure that arise from external or structural sources, rather than by the underlying characteristics of the individuals in the study.

In an RCT, the source of such exogenous variation is explicit: the investigator randomly assigns individuals to different exposure conditions. This ensures that differences in exposure are, on average, unrelated to confounding factors. In observational settings, similar types of variation can sometimes arise naturally or through policy and institutional rules. For example, governmental policy changes in cigarette taxation or the introduction of smoking bans can influence smoking behavior without being directly determined by individual health status or other confounders. In such cases, the resulting variation in exposure may imitate random assignment, even though no formal randomization has taken place. That is, individuals who are affected and not affected by such external changes may be regarded as exchangeable because the mechanism determining exposure is unrelated to the underlying factors that influence the outcome.

This approach to identification has been used in periodontal research, in what is known as an *instrumental variables* design (Figure 8) [36]. This is particularly useful because individuals who smoke and do not smoke differ in many ways that may also affect periodontal health, making simple comparisons between the two groups vulnerable to confounding. By instead relying on exogenous variation in smoking behavior, researchers aim to compare individuals whose smoking status differs for reasons that are less directly related to their underlying health or socioeconomic characteristics. This allows for a comparison between like‐with‐like individuals, facilitating a more credible causal interpretation of the observed differences in periodontal outcomes. Settings such as this are often referred to as *quasi‐experimental* designs or *natural experiments*. However, not all design‐based approaches fall neatly into these categories, and there is ongoing debate as to when a study design is truly considered quasi‐experimental [37]. In general, the key requirement is that a meaningful comparison can be made between groups of individuals who are plausibly exchangeable.

**FIGURE 8:**
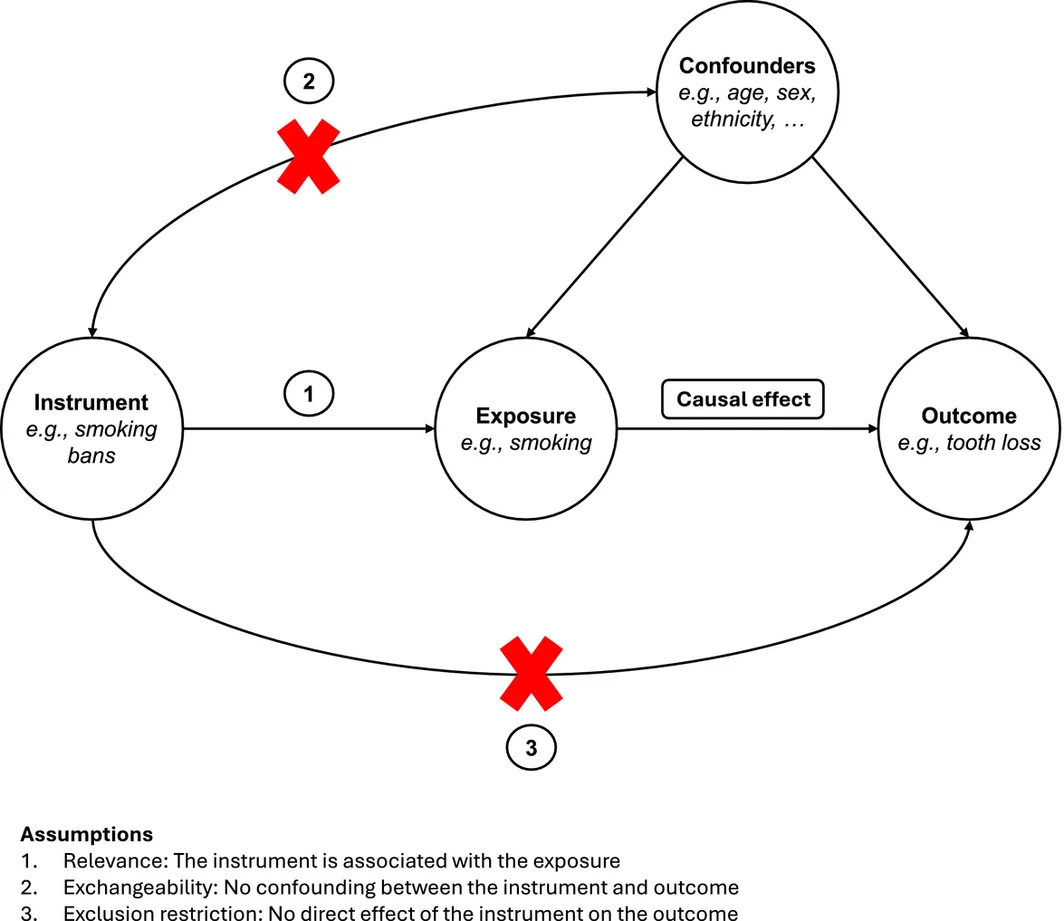
Key assumptions to identify the causal effect in an instrumental variables design. Note the similarity to Figure 6, as RCTs can be viewed as a special case of an instrumental variables design. The double‐headed arrow between the instrumental variable and confounders indicates that the exchangeability assumption is violated regardless of whether the confounders affect the instrument or the instrument affects the confounders.

Consider the example where an investigator is interested in the causal effect of co‐payments on dental attendance. In Japan, individuals receive discounted dental care when they reach 70 years of age. This creates a sharp change in the financial cost of care at a known threshold. However, it is plausible that individuals who are just below and just above this age cutoff (e.g., those aged 68 to 69 compared with those aged 70 to 71) are otherwise broadly comparable in terms of their underlying health status, socioeconomic circumstances, and oral health behavior. Within this narrow range, it is therefore reasonable to regard individuals as approximately exchangeable, with differences in dental attendance arising primarily from the change in copayment policy rather than from underlying differences between the groups. This approach has been used in what is known as a *regression discontinuity* design (Figure 9) [38].

**FIGURE 9:**
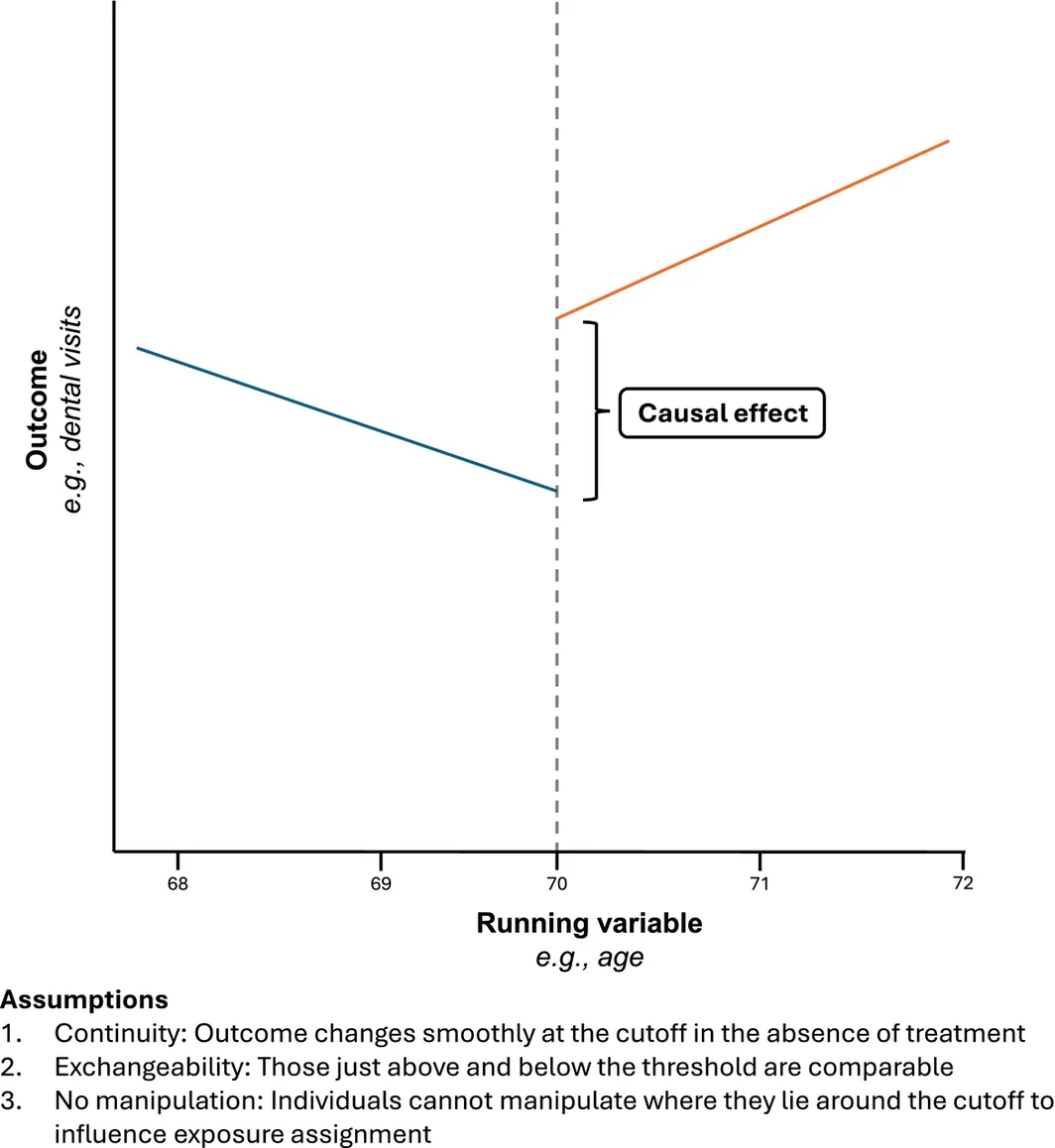
Key assumptions to identify the causal effect in a regression discontinuity design.

Similarly, exchangeability can also be considered not in terms of individuals at a single point in time, but in terms of how groups evolve over time. Two groups may follow similar trajectories in an outcome until one group is exposed to a change that the other does not experience. If these trends would have continued in parallel in the absence of the exposure, then differences in how the outcomes evolve after the intervention can be attributed to the exposure itself. For example, consider a policy change that removes or reduces financial barriers to dental care in one setting but not in another. In Scotland, the introduction of free dental examinations in April 2006 led to changes in patterns of dental attendance compared with other parts of the UK where no such policy change occurred [39]. If the regions would otherwise have followed similar trends in dental care utilization, then the divergence in outcomes after the policy change can be interpreted as evidence of a causal effect. This type of comparison forms the basis of what is known as a *difference‐in‐differences* design (Figure 10).

**FIGURE 10:**
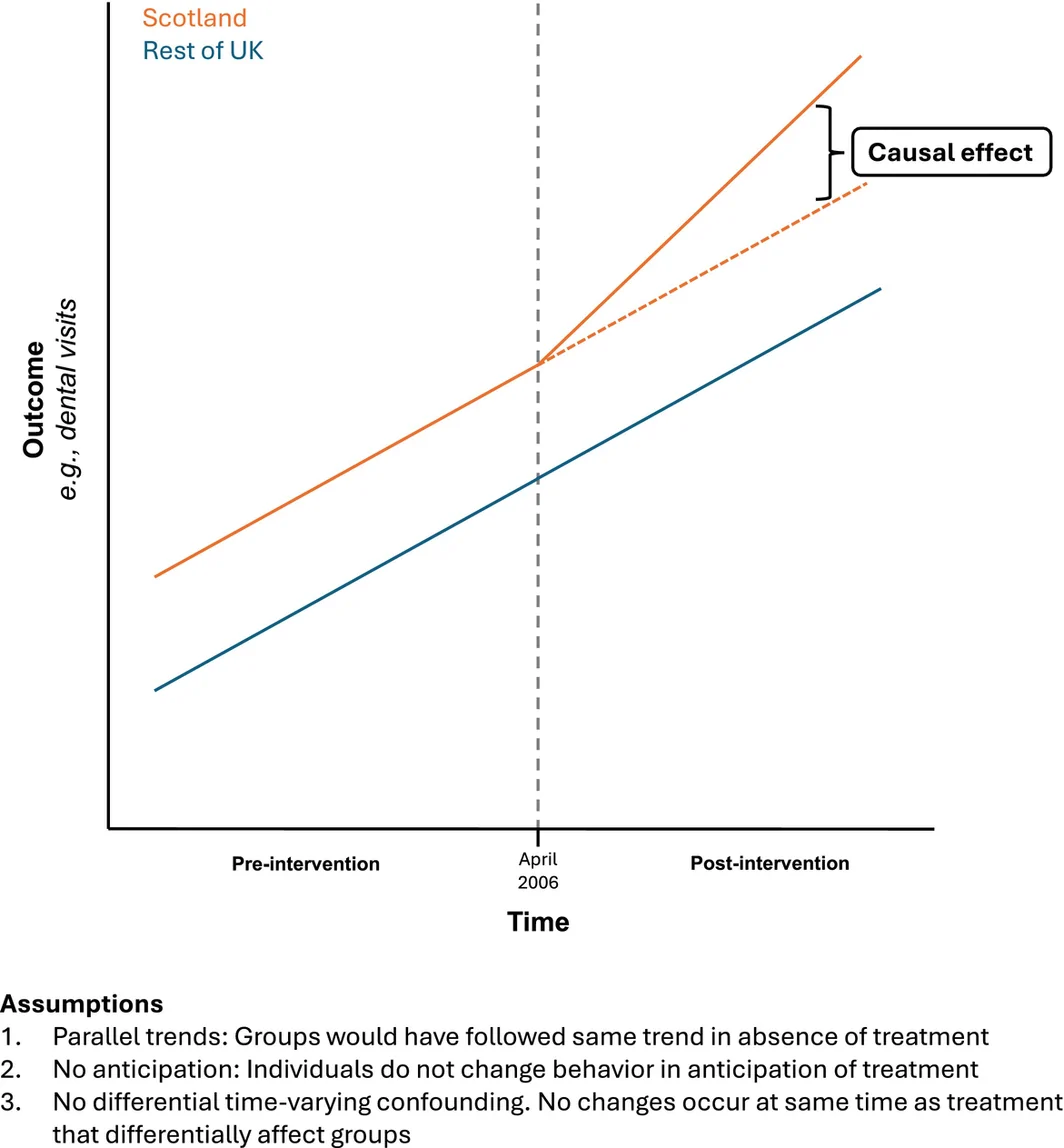
Key assumptions to identify the causal effect in a difference‐in‐differences design. Dashed orange line indicates the assumed trajectory of the exposed group, had they not been exposed.

The aim of this section is not to give an exhaustive discussion of design‐based approaches, but to outline how a credible approach to causal inference is to exploit aspects of the data‐generating mechanisms that facilitate like‐with‐like comparisons.

### 4.3 Adjustment‐Based Identification

One of the most commonly used strategies for identification in observational research is covariate adjustment. The central idea is straightforward; if all variables that jointly influence the exposure and the outcome are measured, then it may be possible to recover the causal effect by comparing individuals who are similar with respect to these variables but differ in their exposure status. Formally, this corresponds to blocking all non‐causal (backdoor) pathways between the exposure and outcome by controlling for an appropriate set of covariates [40, 41].

The underlying identifying assumption is that all confounders of the exposure–outcome relationship are known and measured, and that conditioning on them is sufficient to remove bias. For example, in studies of smoking and periodontitis, investigators may adjust for age, sex, and SEP. If these variables fully capture the common causes of both smoking and periodontal disease, then the adjusted comparison between smokers and non‐smokers can be interpreted causally. However, if relevant confounders are omitted, or if variables affected by the exposure (such as mediators or colliders) are incorrectly included in the adjustment set, bias may be introduced rather than removed. As a result, covariate adjustment is not simply a statistical procedure, but a statement about the assumed causal structure.

This highlights the key distinction between design‐based and adjustment‐based approaches. Adjustment‐based approaches rely on the investigator being able to measure enough confounding variables so that, after accounting for them, the exposure is no longer related to the outcome through confounding pathways. Design‐based approaches, by contrast, aim to relax the assumption that all relevant variables are known and measured, by instead relying on assumptions regarding the nature of the data‐generating process. In this sense, the burden of identification is shifted away from exhaustive covariate measurement and towards assumptions about how the exposure is distributed. That is not to say design‐based identification is always superior to adjustment‐based approaches, or *vice versa*. Rather, these two approaches differ in where they place their assumptions and how they are justified. It is entirely possible for a design‐based approach to leverage highly plausible sources of exogenous variation, but for these assumptions to later be questioned [42, 43]. Similarly, it is possible to obtain invalid causal inferences when using adjustment‐based approaches where important confounders have gone unmeasured or mismeasured.

Regression is not the only approach for adjustment‐based identification. A summary of key study designs and analytic methods used in causal inference, along with where readers can find guides to each method, is presented in Table 2.

**TABLE 2:** Study designs and analytic approaches used in causal inference.

Study design/analytic method	Description	Introductory guide
Bounds approaches	Provide upper and lower limits on causal effects when assumptions are partially violated.	[44]
Difference‐in‐differences	Compares changes in outcomes over time between treated and untreated groups in (at least) two contexts.	[45]
Doubly robust	Combine two approaches, modeling both the outcome and exposure, so that the causal effect estimate remains consistent if either model is correctly specified.	[46]
E‐values	Quantify the strength of unmeasured confounding needed to explain away an observed association.	[47]
Falsification tests	Test for causal validity by checking for effects in settings where no effect is expected.	
Fixed‐effects/Within‐individual	Exploit within‐person variation in exposure over time by comparing each individual to themselves.	[48]
G‐computation	Uses the observed data to estimate counterfactual outcomes by modeling the full distribution of the variables.	[49]
Instrumental variables	Uses a variable that affects the exposure, but only affects the outcome through the exposure.	[50]
Interrupted time series	Examines the effect of an intervention by comparing trends before and after its implementation.	[51]
Matching	Matches treated and untreated individuals with similar covariates to mimic randomization.	[52]
Mendelian randomization	Uses genetic variants as instrumental variables to assess causal effects of modifiable exposures.	[53]
Multivariable regression	Adjusts for measured covariates to estimate the effect of an exposure on an outcome.	[54]
Natural experiments	Uses naturally occurring variation as if it were random assignment.	[55]
Negative controls	Uses an exposure or outcome that should not be causally related to detect bias.	[56]
Propensity scores	Summarize covariates into a single score to balance treated and untreated groups.	[57]
Randomized controlled trial	Random assignment of individuals to treatment and control groups so that any difference in outcomes is attributable to the treatment effect.	[58]
Regression discontinuity	Exploits a cutoff or threshold to compare units just above and below the threshold.	[59]
Sibling/twin studies	Compares siblings or twins to control for shared genetic and environmental confounding.	[60]
Structural equation models	Specifies relationships among multiple variables including latent constructs.	[61]
Synthetic controls	Constructs a weighted combination of control units to approximate the treated unit's counterfactual.	[62]
Targeted maximum likelihood estimation	Doubly robust approach that uses a two‐stage “targeting” approach to identify the optimum bias‐variance tradeoff for the target estimand.	[63]
Target trial emulation	Frames observational analyses as if they were randomized trials by explicitly specifying eligibility, treatment strategies, assignment, follow‐up, and causal contrasts.	[30]

*Note:* Where possible, non‐technical (i.e., non‐mathematical) introductory guides have been cited.

## 5 Analysis

Once a causal effect has been defined and identified, the final step is to translate this into an empirical estimate using data. This stage is referred to as *analysis*. It is important to be clear about its role: analysis does not establish causality, nor does it determine what the causal effect is. Those decisions are made earlier, at the stages of defining the question and establishing identification. Analysis is simply the process of computing the identified quantity (which under the proposed assumptions, has a causal interpretation) from the observed data.

Different estimands correspond to different causal contrasts (e.g., effects in specific subgroups versus the overall population), and these in turn require different analytical strategies for their estimation. In this sense, the estimand effectively determines what must be estimated from the data, and therefore constrains and guides the appropriate choice of analysis.

### 5.1 Analysis Following Design‐Based Identification

Analysis following design‐based identification tends to be relatively simple, because much of the work required for causal interpretation has already been done at the design stage. If the study design has successfully created a comparison between groups that are plausibly exchangeable, the role of analysis is primarily to estimate and describe the resulting difference in outcomes. For example, in a randomized controlled trial, analysis may involve little more than comparing the average outcome between treatment groups. Similarly, in quasi‐experimental settings such as instrumental variables studies, the statistical model is often relatively straightforward because identification comes from the structure of the design itself, rather than from adjustment for a large number of measured confounders.

This does not mean that analysis is unimportant. Investigators can sometimes evaluate whether the assumptions made at the design stage are plausible using statistical analysis. For example, the relevance assumption in an instrumental variables analysis can be examined by assessing the strength of the association between the instrumental variable and the exposure of interest. Similarly, in a regression discontinuity design, investigators may examine whether individuals just above and below the threshold are comparable with respect to baseline characteristics. These checks do not prove that the assumptions hold, but they can increase or decrease confidence in the credibility of the design.

### 5.2 Analysis Following Adjustment‐Based Identification

When identification is achieved through covariate adjustment, the goal of analysis is to compare individuals who are similar with respect to the set of identified confounding variables. In practice, this is most commonly implemented using *multivariable regression* models [64, 65]. For example, in a study of smoking and periodontitis, a regression model might include smoking status as the exposure and adjust for variables such as age, sex, SEP, and oral hygiene behaviors. The coefficient for smoking is then interpreted as the estimated causal effect, provided the adjustment set is sufficient to block confounding pathways.

Regression modeling is only one way of implementing adjustment‐based identification. Another approach is through *stratification*, where individuals are compared within levels of a confounder. For example, smokers and non‐smokers may be compared separately within age groups or socioeconomic strata. This makes the comparison more like‐with‐like by ensuring that exposure groups are compared within more homogeneous subgroups. Another common approach is *matching*, where exposed individuals are paired with unexposed individuals who have similar values of important confounding variables. For instance, a smoker may be matched to a non‐smoker of similar age, sex, and SEP. The analysis is then based on these matched comparisons rather than the full unmatched sample. Although some matching procedures involve explicit pairwise comparisons between treated and untreated individuals, the overall goal remains estimation of a population‐level causal effect rather than inference about the matched pairs themselves [66].

A related method is the use of *propensity scores*, which summarize multiple confounding variables into a single quantity—the propensity score—representing the probability of receiving the exposure, given the observed covariates [57]. Individuals with similar propensity scores are then compared using matching, stratification, or weighting. This can be particularly useful when many confounders must be considered simultaneously. As with the previous adjustment‐based approaches, propensity score methods correct for measured and not unmeasured confounding.

There are several other advanced analytical approaches, such as structural models, doubly robust methods, and causal machine learning [15, 46, 67]. Although the statistical techniques and underlying computation may differ in complexity, the principle is unchanged: the relevant confounders must all be known and measured.

### 5.3 Joint Approaches

The dichotomization of approaches into design‐based versus adjustment‐based is useful for explanation, but may not be exact in practice. Many studies combine elements of both approaches, using features of the study design to improve exchangeability while also applying statistical adjustment to account for remaining differences between groups.

For example, an instrumental variable analysis may still include adjustment for baseline covariates to improve precision and reduce residual imbalance between the groups being compared. Similarly, a regression discontinuity design may adjust for variables measured around the threshold, even when identification is primarily justified by the cutoff itself. Likewise, some adjustment‐based approaches can incorporate design‐like thinking. Matching methods explicitly attempt to construct groups that resemble those that might have been created through randomization by restricting comparisons to individuals who are sufficiently similar with respect to measured confounders. Because matching typically relies on the assumption that all relevant confounders are known and measured, it is useful to categorize it here as an adjustment‐based approach (though some matching strategies, such as within‐sibling comparisons, can also address unmeasured confounding that is stable over time). However, its logic is also strongly aligned with the design‐based goal of creating a like‐with‐like comparison between exposed and unexposed groups. This reflects the broader principle that methods are rarely isolated procedures, but tools used together to strengthen the plausibility of causal interpretation i.e., it is useful to think of design‐based and analysis‐based identification as covering a continuum of approaches.

## 6 What Has Been Inferred?

Across all approaches, a final component of analysis is quantifying uncertainty. This is typically done using standard errors, confidence intervals, or Bayesian posterior distributions. These measures describe how much the estimate would vary if the study were repeated under the same assumptions. However, it is important to distinguish this from a deeper form of uncertainty: uncertainty about whether the identifying assumptions themselves are correct. Statistical methods can quantify random sampling variability, but they cannot resolve whether confounding has truly been removed, whether the instrumental variable is valid, or whether the regression discontinuity assumptions hold. This type of uncertainty must be addressed through study design, subject‐matter knowledge, and transparent reasoning.

So, what, then, is *inference*? In statistics, “inference” may often be used in a narrower sense to mean quantifying uncertainty around an estimate. However, in its broader scientific sense, inference is the process of moving from observed data to a conclusion about an unobserved causal quantity. This distinction is important because it clarifies that causal inference is not a single step, but a chain of reasoning. At one level, *statistical* inference is performed, which concerns how much an estimate would vary due to random sampling. But in *causal* inference, there is a second and more fundamental level. This concerns whether the quantity being estimated corresponds to a meaningful causal effect in the first place. It depends not on repeated sampling, but on whether the assumptions required for identification are credible.

These two layers of inference are often conflated, but they address fundamentally different questions. Statistical inference operates within a model; causal inference depends on whether the model itself is appropriate in the first place. This separation also clarifies why highly precise estimates can still be misleading. A narrow confidence interval does not guarantee that the estimate is close to the true causal effect because it does not account for violations of identifying assumptions. In other words, it is possible to be very certain about the wrong answer if the causal structure has been misspecified. A useful way to summarize this distinction is to think of causal inference as involving two linked but distinct uncertainties. The first is uncertainty due to sampling variability, which is reducible with larger datasets. The second is uncertainty due to identification, which is not reducible by data alone and instead depends on scientific judgment, study design, and external knowledge.

Therefore, inference, in its full sense, requires not only statistical machinery but also a defensible chain of assumptions connecting data to the causal question of interest.

## 7 Selection Bias

The discussion up to this point has largely focused on confounding, and this is because confounding is often the most prominent and intuitively accessible threat to causal inference. However, confounding is only one source of bias that can threaten causal interpretation. Even when confounding has been carefully addressed, other mechanisms can still distort causal estimates. Therefore, it would not be correct to equate valid causal inference with simply having adequate control for confounding. In fact, some persistent difficulties in applied causal inference arise not from confounding, but from issues related to how individuals are selected into a study [68].

### 7.1 Collider Bias

It is helpful to first recall the earlier discussion of collider variables. A collider is a variable that is itself influenced by two other variables, and conditioning on a collider can induce an association between its causes even when none exists in the underlying population. One form of such conditioning on a collider arises when inclusion in a study depends on variables that are themselves affected by both the exposure and the outcome. In such settings, selection into the analytic sample effectively acts as conditioning on a collider. As a result, associations may be induced between exposure and outcome even if no such relationship exists in the target population, resulting in *collider bias*.

For example, suppose a study of periodontal disease is conducted only among individuals who attend dental clinics and have a recorded periodontal examination. Attendance is not random; it may depend both on an individual's underlying oral health and on factors such as SEP or health‐seeking behavior. If both the exposure and outcome (or their determinants) influence the probability of being observed, then conditioning on “being in the study” can introduce an association between variables that are otherwise unrelated in the full population. In this setting, the observed dataset no longer reflects the causal structure of the target population. This may have implications for downstream analyses, an example of which is presented in Figure 11.

**FIGURE 11:**
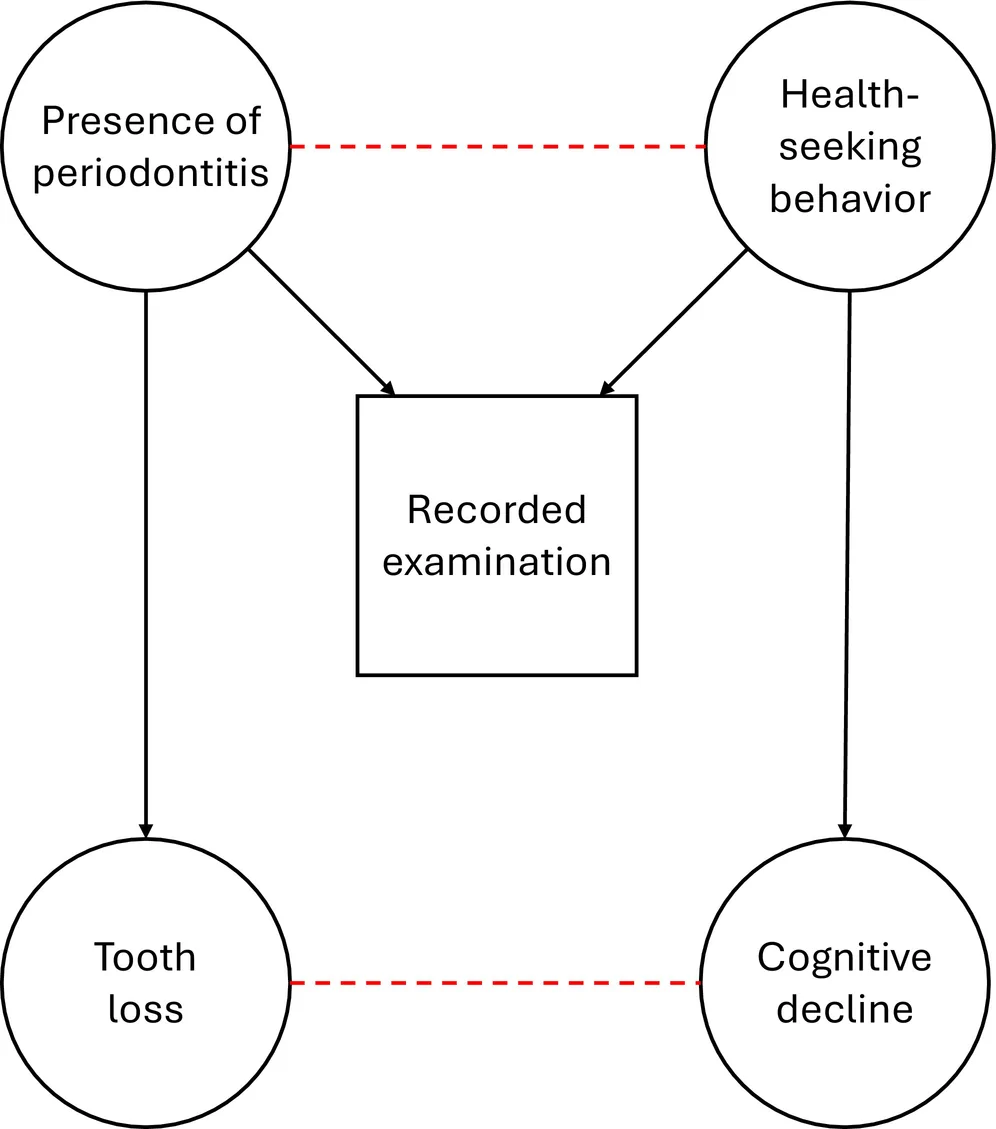
Directed acyclic graph representing collider bias in an analysis evaluating the effect of tooth loss on cognitive decline. Having a recorded examination is a common effect (i.e., collider) of periodontal disease status and health‐seeking behavior. Square nodes denote variables that were conditioned on e.g., by restricting to individuals with periodontitis. Dashed red lines denote correlation between variables that are not necessarily causal. Restricted the analysis to individuals with a recorded examination induces a correlation between periodontitis and health‐seeking behavior and, therefore, also between tooth loss and cognitive decline, even if no causal effect is present. For clarity of exposition, not all plausible causal arrows are shown, as their inclusion would not alter the underlying collider bias structure represented in the graph.

### 7.2 Effect Modification

An effect modifier is a variable that changes the magnitude of a causal effect across individuals or subgroups. When selection into a study depends on such variables, the resulting sample may contain a different mixture of effect sizes than the target population. This can distort the observed average effect, even when confounding has been adequately addressed and no collider structure is present. Effect modification is related, but not equivalent, to the concept of statistical interaction [69].

For example, consider smoking and periodontitis, but imagine that the effect of smoking is known to be stronger in individuals with poor oral hygiene. If the study sample is only made up of individuals who regularly attend the dentist, then it may disproportionately include individuals with good oral hygiene, since they are more likely to attend routinely. As a result, the estimated effect of smoking may appear smaller than it truly is in the wider population, simply because individuals in whom the effect is strongest are under‐represented.

## 8 Representativeness and Transportability

The concept of *representativeness* refers to the extent to which the study sample reflects a specified target population. It is useful to distinguish between a *source population*, from which the study sample is drawn, and a *target population*, to which causal conclusions are intended to apply. A study sample may be unrepresentative of the source population, yet still be fully appropriate for making causal inferences about a well‐defined target population. For example, if a drug is intended to be used only in women of pre‐menopausal age, then one would not expect the study sample to be representative of all individuals in the broader source population across all age groups and sexes. In fact, representativeness of this kind is not only unnecessary, but often undesirable. It may reduce the precision of causal estimates and introduce heterogeneity that is irrelevant to the causal question of interest. The scientific value of a study is not determined by how closely it reproduces the distribution of a population, but by whether it enables valid comparisons that elucidate underlying causal relationships [70]. This distinction also clarifies why representativeness is not, in itself, a requirement for causal inference, even though it is central to some descriptive objectives such as estimating disease prevalence or incidence.

This naturally leads into discussion around the concept of *transportability*, which concerns whether causal effects estimated in one population can be generalized to another. Even if a causal effect is correctly identified in a study sample, it may not translate to a different target population if the selection mechanism differs in ways that are related to colliders or effect modifiers. For instance, a periodontal study conducted in a specialist clinic population may not generalize to the general population if clinic attendees differ systematically in disease severity or health behavior. In such cases, the issue is not only whether the internal causal estimate is valid, but whether it applies outside the selected sample [68]. This is something which investigators need to evaluate based on substantive knowledge.

## 9 Concluding Remarks

This article has set out to provide a structured overview of how investigators may approach causal inference. Robust causal inference typically proceeds from a clearly defined causal question: what effect is being estimated, in which population, and under what hypothetical interventions?

No single method for causal inference is universally superior; rather, each approach requires careful consideration of what must be believed for the resulting conclusions to be valid. Directed acyclic graphs are a useful way of making these assumptions explicit, but they do not themselves generate causal knowledge; their validity depends entirely on the assumptions used to construct them, which are ultimately based on subject‐matter knowledge. Accordingly, specifying an appropriate DAG and deriving a valid adjustment set is not a mechanical procedure, but a substantive scientific exercise. Taken together, this highlights that causal inference is less about choosing the “best” statistical technique and more about choosing a set of assumptions that are defensible in the context of the scientific question. The risk in practice is often not that investigators believe assumptions are unnecessary, but that the use of a particular statistical method or analytical approach can create the impression that causal conclusions follow automatically from the method itself.

For instance, investigations into the relationship between periodontal and systemic health often rely on observational data analyzed using multivariable regression models. In these settings, the key identifying assumption is that of no unmeasured confounding—that all variables jointly affecting both periodontal and systemic outcomes have been appropriately measured and adjusted for. This immediately raises a substantive question: how plausible is it, in practice, that all relevant confounders of such complex systems have been captured?

There are many additional considerations in applied analyses that go beyond the scope of this review, including measurement error and the decomposition of effects into mediating pathways. Likewise, issues such as missing data, statistical power, sample size, and precision affect the level of uncertainty around an estimate, and therefore its informativeness. Although greater precision increases statistical confidence in the estimated effect, it does not alter its causal validity once the necessary identifying assumptions are satisfied. Each of these topics introduces additional layers of complexity that can affect both estimation and interpretation, often requiring further assumptions beyond those already discussed.

Ultimately, causal inference methods differ in where they place assumptions, but none can remove them entirely. The central task for researchers is therefore to articulate a clear causal question, make the underlying assumptions explicit, and critically evaluate their plausibility. When this is done transparently, causal claims become easier to interpret, critique, and build upon, regardless of the specific method used.

## Funding

NZB is supported by the Wellcome Trust (322777/Z/24/Z). SB is supported by the Wellcome Trust (225790/Z/22/Z). NZB and SB are members of the Medical Research Council Biostatistics Unit (MC_UU_00040/01).

## Disclosure

AI Statement: This manuscript used NLP for proofreading an entirely human‐generated text.

## Conflicts of Interest

The authors declare no conflicts of interest.

## Data Availability

Data sharing not applicable to this article as no datasets were generated or analysed during the current study.
